# Physical activity alters the effect of genetic determinants of adiposity on hypertension among individuals of European ancestry in the UKB

**DOI:** 10.1111/sms.14636

**Published:** 2024-04-26

**Authors:** Chukwueloka Hezekiah, Alexandra I. Blakemore, Daniel P. Bailey, Raha Pazoki

**Affiliations:** ^1^ Cardiovascular and Metabolic Research Group, Division of Biomedical Sciences, Department of Life Sciences, College of Health, Medicine and Life Sciences Brunel University London London UK; ^2^ Department of Mental Health, Faculty of Health, Science, Social Care and Education Kingston University Surrey UK; ^3^ Department of Life Sciences, Centre for Cognitive Neuroscience, College of Health, Medicine and Life Sciences Brunel University London London UK; ^4^ Department of Metabolism Digestion and Reproduction, Imperial College London London UK; ^5^ School of Medicine University of Ireland Galway Ireland; ^6^ Centre for Physical Activity in Health and Disease, College of Health, Medicine and Life Sciences Brunel University London Uxbridge UK; ^7^ Division of Sport, Health and Exercise Sciences, Department of Life Sciences Brunel University London Uxbridge UK; ^8^ Department of Epidemiology and Biostatistics, School of Public Health Imperial College London London UK

**Keywords:** African, European, genetic risk, hypertension, obesity, physical activity

## Abstract

Hypertension is a leading risk factor for cardiovascular disease and is modulated by genetic variants. This study aimed to assess the effect of obesity genetic liability and physical activity on hypertension among European and African ancestry individuals within the UK Biobank (UKB). Participants were 230 115 individuals of European ancestry and 3239 individuals of African ancestry from UKB. Genetic liability for obesity were estimated using previously published data including genetic variants and effect sizes for body mass index (BMI), waist‐hip ratio (WHR) and waist circumference (WC) using Plink software. The outcome was defined as stage 2 hypertension (systolic blood pressure ≥ 140 mmHg, diastolic blood pressure ≥90 mmHg, or the use of anti‐hypertensive medications). The association between obesity genetic liability and the outcome was assessed across categories of self‐reported physical activity using logistic regression. Among European ancestry participants, there was up to a 1.2 greater odds of hypertension in individuals with high genetic liability and low physical activity compared to individuals with low genetic liability and high physical activity (*p* < 0.001). In individuals engaging in low levels of physical activity compared with moderate/high physical activity, the effect of BMI genetic liability on hypertension was greater (*p*_interaction_ = 0.04). There was no evidence of an association between obesity genetic liability and hypertension in individuals of African ancestry in the whole sample or within separate physical activity groups (*p* > 0.05). This study suggests that higher physical activity levels are associated with lower odds of stage 2 hypertension among European ancestry individuals who carry high genetic liability for obesity. This cannot be inferred for individuals of African ancestry, possibly due to the low African ancestry sample size within the UKB.

## 1 INTRODUCTION

Hypertension is a major worldwide health challenge, accounting for an estimated 10.4 million deaths annually.^1^ Approximately 1.3 billion adults suffered from hypertension in 2019 (World Health Organization, 2023). An estimated 31% of European ancestry adults suffer from hypertension, with only 49% of the hypertensive cases having their hypertension under control.^2^ The prevalence of hypertension (45%) is higher in African ancestry adults, with effective control being less prevalent (39% of hypertension cases).^2^

A number of factors are associated with hypertension risk including obesity,^3^, ^4^, ^5^ lifestyle behaviors such as physical inactivity,^6^, ^7^, ^8^, ^9^ and genetics.^10^ Obesity increases hypertension,^10^, ^11^ while physical activity, such as aerobic and resistance exercise, effectively reduces hypertension.^6^, ^12^ There is evidence that physical activity and obesity interacts in modifying the risk of hypertension. For example, self‐reported physical activity reduced the effect of obesity on hypertension risk by 37% in a cohort study of 13 715 Australian women.^13^ A similar result has also been demonstrated among men.^14^ Therefore, evidence suggests that physical activity may reduce the effect that obesity has on hypertension.

A genetic susceptibility is recognized for hypertension as numerous genetic factors are linked to high blood pressure.^15^ For example, obesity genetic liability has an increasing effect on hypertension^10^ and improves performance of hypertension prediction models.^16^, ^17^ A combination of physical activity and low obesity genetic liability reduces obesity,^18^, ^19^ but it is unclear if physical activity affects the relationship between obesity genetic liability and hypertension. Understanding the interplay between these different factors (i.e. obesity, physical activity, and genetic factors) in relation to hypertension risk will further knowledge in relation to potential diagnostic and intervention targets for promoting public health. The aim of this study, therefore, was to investigate the effect of obesity genetic liability on hypertension across different levels of physical activity within European and African ancestry samples of the UK Biobank (UKB).

## 2 METHODS

### 2.1 Ethical approval

The UKB obtained ethical approval from the North West Multi‐centre Research Ethics Committee as a Research Tissue Bank approval. All participants gave informed consent. This research is performed using UKB data under application number 60549. Ethical approval for the current analysis to work on secondary data from the UKB was obtained from Brunel University London's College of Health, Medicine and Life Sciences Research Ethics Committee (reference 27 684‐LR‐Jan/2021–29 901‐1).

### 2.2 Study population

The UKB is a large population‐based cohort study established in 2006 to enable comprehensive investigations of genetic, environmental and lifestyle determinants of health, morbidity, and mortality. The study includes >500 000 participants who live in the United Kingdom aged between 40 and 69 years at the time of recruitment.^20^

### 2.3 Genotyping and imputation

DNA extraction and genotyping were undertaken by the UKB. Detailed information regarding genotyping and imputation has been provided elsewhere.^21^, ^22^, ^23^ In brief, participant blood samples were collected at the UKB assessment centre and genetic data of 488 377 participants was extracted. The first batch of the participants (*n* = 49 950) were genotyped using a comparable Applied Biosystems™ (UK BiLEVE Axiom™ Array by Affymetrix) consisting of 807 411 markers. The remainder of the samples (*n* = 438 427) were genotyped using an Applied Biosystems™ (UKB Axiom™ Array) including 825 927 markers designed to capture short insertions and deletions (indels) and Single Nucleotide Polymorphisms (SNPs).^21^

To maximize the use of haplotypes with British and European ancestry for imputation, genotype imputation used three reference panels (Haplotype Reference Consortium, UK10K, and 1000 Genomes phase 3). Genotype imputation was performed by the UKB using the IMPUTE 4 programme. Genetic principal components were computed by the UKB to account for population stratification.^21^

### 2.4 Sample for analysis

The current study was performed using two subsets of unrelated individuals of European and African ancestry within the UKB. Participants were excluded (Figure S1) if they had withdrawn consent from UKB (*n* = 80). Genetic data were available for 488 377 individuals. After merging genetic and phenotype data, 487 206 individuals remained. Participants who were first‐ and second‐degree relatives (kinship coefficient threshold <0.1768) for at least one other UKB participant (*n* = 26 124) were excluded. Participants were also excluded if (1) they self‐reported to be of non‐European and non‐African ancestry (*n* = 19 743), (2) their self‐reported sex did not match their genetic sex (*n* = 330), and (3) they were pregnant or unsure of their pregnancy status at baseline (*n* = 567). In addition, participants who did not declare their smoking status (*n* = 238), or participants with missing data in their *pack‐years of smoking* (*n* = 67 115), and current or previous smokers (*n* = 1065) for whom zero *pack‐years of smoking* was calculated, were excluded. This could have occurred due to missing values in (1) the age of smoking initiation or cessation, or (2) the number of cigarettes they smoked per day.

This study further excluded 13 907 individuals who were unsure about their dietary intake of fish, meat, fruits or vegetables and 77 792 individuals with missing data in the main study covariates (see below in Assessment of covariates). In addition, participants who (1) used cholesterol‐lowering medication (*n* = 46 679), (2) whose self‐reported ancestry did not match their genetic ancestry (*n* = 173), or (3) did not declare drinking status (*n* = 98) were also excluded. The sample was then divided into European (*n* = 230 136) and African (*n* = 3239) ancestry subsets using self‐reported ethnicity data. Participants who withdrew their consent after the analysis was completed were also excluded from the European sample (*n* = 21) and the results were adjusted. The final European sample used in the analysis was 230 115 (Figure S1).

### 2.5 Phenotypic data

Following informed consent, a broad selection of phenotypic information was collected during baseline assessment in the UKB (the first visit for each participant, which took place between 2006 and 2010). Data were collected from answers provided during interviews and using touch‐screen questionnaires. This included socio‐demographic, health and lifestyle‐related information. Participants also completed a range of physical and anthropometric measurements during the baseline assessment. They provided saliva, urine, and blood samples, which were used for various proteomic, genetic and metabolomic analyses.^24^

### 2.6 Blood pressure and definition of hypertension

During the baseline assessment in the UKB, two automated or manual systolic blood pressure (SBP) and diastolic blood pressure (DBP) readings were taken after 2 min rest. An Omron HEM 7015‐T automated digital blood pressure device was used for reading the automated blood pressure data. The measurements were taken with an appropriately sized cuff on each participant's left upper arm (the right arm was used where it was not practical to use the left arm). A manual sphygmomanometer was used to measure blood pressure manually in instances where the automated blood pressure device could not be used. All blood pressure values were measured in mmHg. These baseline measures were used to determine prevalent hypertension cases in the present study. This included averaging the SBP and DBP readings. For participants with one manual and one automated reading, the average SBP and DBP were calculated using those values only. This approach aligns with methods used in our previous publications.^25^ To minimize the effects of blood pressure medication on the study outcomes, participants on blood pressure‐lowering medications (*n* = 91 785) had 15 and 10 mmHg added to their average SBP and DBP readings, respectively.^26^ Stage 2 hypertension was defined in line with the American Heart Association guidelines, that is, SBP ≥140 or DBP ≥90 mmHg.^27^ In addition, participants using antihypertensive medication were considered as stage 2 hypertensive.

### 2.7 Physical activity categories

Within the UKB, an adapted version of the short International Physical Activity Questionnaire (IPAQ) was used to assess physical activity.^28^ Information on the frequency and duration of walking, moderate and vigorous physical activity was collected. Cassidy and colleagues^29^ used the IPAQ data processing guidelines to produce the Metabolic Equivalent of Task (MET) minutes per week of physical activity by multiplying the duration of walking, moderate and vigorous physical activity by 3.3, 4.0 and 8.0 METs, respectively. These were summed to provide total physical activity in MET min/week.^30^ The data were returned to the UKB by Cassidy and colleagues,^29^ which were used in the present study to categorize physical activity into^1^ low (total physical activity <600 MET‐min/week) and^2^ moderate (≥600 MET‐min/week) or high (≥3000 MET‐min/week) physical activity.^30^ We grouped moderate and high physical activity together as the World Health Organization recommends engaging in moderate or high physical activity to gain health benefits.^31^

### 2.8 Assessment of covariates

Covariates were selected based on established risk factors for cardiovascular disease. These covariates align with those commonly utilized in previous related studies.^25^ Participants were asked to select their daily quantity of dietary consumption of *vegetables* (*cooked* and *raw*), *fruit* (*fresh* and *dried*), *oily fish* and *meat* (*processed* and *unprocessed*, including *poultry*, *lamb*, and *pork*).

*Smoking status* in the UKB was assessed using a self‐reported question categorizing smoking status into current, past, and never smoking. *Pack‐years of smoking* was available for European (*n* = 230 115) and African (*n* = 3239) ancestry samples. *Pack‐years of smoking* was assessed as the number of cigarettes smoked per day divided by twenty (as the average pack size), multiplied by the number of years smoking.^32^ The number of years smoking was calculated by subtracting the age at which the participant started smoking from the age they stopped smoking. In this study, current or previous smokers with any values in their *Pack‐years of smoking* were categorized as smokers. Never smokers were considered as participants who reported never smoked and had zero values in *Pack‐years of smoking*.

Additional covariates for the analysis included self‐reported alcohol intake status (current, past and non‐drinkers) and low‐density lipoprotein (LDL) cholesterol measured by enzymatic protective selection analysis on a Beckman Coulter AU5800. Further details of quality control and sample preparation for the UKB biomarker data have been published previously.^33^

### 2.9 Obesity genetic liability

In the current study, obesity genetic liability was generated using previously reported genetic variants for European and African ancestry individuals. These variants were associated with body mass index (BMI),^34^, ^35^ waist‐hip ratio (WHR),^36^, ^37^ or waist circumference (WC).^36^, ^37^ Within the European ancestry sample, a list of 155 SNPs with their weights (β‐coefficients) from a GWAS performed by Winkler and colleagues^34^ was used to estimate the BMI genetic liability. β‐coefficients for WHR SNPs (*n* = 27) and WC SNPs (*n* = 41) were obtained from Shungin and colleagues^36^ and were used to estimate WHR and WC genetic liabilities. To estimate BMI genetic liability for individuals of African ancestry, a list of seven SNPs with β‐coefficients from Ng and colleagues^35^ was used. To estimate WHR and WC genetic liability in African ancestry, a list of three WHR and two WC SNPs with β‐coefficients were used from previously published GWAS of WHR and WC in this population group.^37^

As part of the SNP selection process, linkage disequilibrium (LD) pruning was performed on the final list of SNPs using UKB individual‐level data. Consequently, SNPs that (1) did not reach a GWAS significance threshold of *p* < 5 × 10^−8^, (2) had Minor allele frequency <0.01, and (3) were dependent to other SNPs as demonstrated by linkage disequilibrium (LD) parameter (*R* ^2^ > 0.1), were excluded. The final list of SNPs used in the analyses are presented in Tables S1 and S2.

Using Plink v1.9,^38^ genetic liabilities were calculated using the Plink function that automatically multiplies previously estimated effects of each genetic variant on obesity phenotype (i.e. BMI, WHR, and WC) by the number of risk alleles carried by each UKB participant on the respective SNPs associated with each phenotype. Consequently, Plink sums the products across all SNPs to produce overall weighted genetic liability for BMI, WHR and WC for each participant. The *base* package in R was used to standardize the genetic liabilities for each participant by subtracting the average genetic liability within the sample from the participant's genetic liability. The resulting score was divided by standard deviation of the genetic liability within the sample.

### 2.10 Data presentation and statistical analysis

To identify genetic distance of the participants (measure of the genetic differences between individuals), a cluster analysis was conducted using the K‐means algorithm.^39^ The K‐means algorithm requires a parameter specifying the number of clusters (K).^40^ A ‘K’ value of seven was assigned according to the number of categories within the UKB self‐reported phenotypic variable, *ethnic background* (Table S3). The genetically derived clusters were compared with the self‐reported ancestries to identify participants whose self‐reported ancestry did not match with the genetically derived ancestry.

Logistic regression was used to estimate the odds of stage 2 hypertension per unit increase in the ancestry‐specific derived standardized obesity genetic liability (BMI, WHR, and WC). The crude odd ratios (ORs), minimally adjusted ORs (adjusted for age and sex), and fully adjusted ORs (adjusted for age, sex, daily alcohol intake, pack‐years of smoking, daily fruit and vegetable intake, meat intake, fish intake, and LDL cholesterol) were calculated. This was estimated for the whole sample and within separate physical activity categories (low and moderate/high). The combined effect of obesity genetic liability and physical activity was examined by comparing the odds of stage 2 hypertension in each combined category with the reference group (low genetic liability and moderate/high physical activity).

For genetic liabilities that demonstrated a statistically significant association with stage 2 hypertension within the whole sample, an interaction test was performed to identify if physical activity group modified the effect of genetic liability on stage 2 hypertension. A sensitivity analysis was performed using European ancestry‐derived SNPs in the African ancestry sample. Missing variables (covariates) were imputed to minimize missing data within the smaller African ancestry sample size. Multiple Imputation by Chained Equation (*MICE*) package v3.13.0 in R was used to create multiple predictions for each missing value (LDL cholesterol, pack years of smoking, and IPAQ physical activity group).^41^ The threshold for missingness was <5% for each row. Predictor variables (age, sex, smoking status, alcohol intake, insomnia, education, genetic principal components, current employment, fruit and vegetable intake) were selected based on their relevance to the research question and availability in the dataset. Ten iterations of the MICE algorithm were performed. Analysis for sub‐groups of physical activity were repeated using the imputed data. The post imputation sample size that was used for sensitivity analysis included 4942 African ancestry participants. Statistical significance was accepted if the type 1 error (*p*‐value) was below 0.05. All statistical analyses were implemented in R v4.0.0.^42^

## 3 RESULTS

### 3.1 European ancestry results

This study included 230 115 European ancestry participants from the UKB (Table 1; Table S4). The prevalence of hypertension differed significantly between the low physical activity group (46.5%) and the moderate/high physical activity group (45.70%). Participants with low physical activity had a significantly higher BMI (mean = 28.0 kg/m^2^; SD = 5.19) compared to the moderate/high physical activity group (mean = 26.6 kg/m^2^; SD = 4.35).

**TABLE 1:** Baseline characteristics of the study sample.

Characteristics	African			European		
	Physical activity level		*p*‐Value for difference in physical activity level*	Physical activity level		*p*‐Value for differences in physical activity level*
	Low (*n* = 700)	Moderate/High (*n* = 2539)		Low (*n* = 41 604)	Moderate/High (*n* = 188 511)	
Age (Years)						
Mean (SD)	49.7 (7.2)	50.9 (7.5)	<0.001	54.9 (7.7)	55.5 (8.1)	<0.001
Median [Min, Max]	49.0 [40, 70]	49.0 [40, 70]		55.0 [40, 70]	56.0 [38, 73]	
Sex						
Female, *n* (%)	419 (59.9%)	1540 (60.7%)	0.74	23 099 (55.5%)	105 178 (55.8%)	0.31
Male, *n* (%)	281 (40.1%)	999 (39.3%)		18 505 (44.5%)	83 333 (44.2%)	
Stage 2 hypertension cases^a^						
No, *n* (%)	353 (50.4%)	1226 (48.3%)	0.34	22 261 (53.5%)	102 314 (54.3%)	4.51 × 10^−3^
Yes, *n* (%)	347 (49.6%)	1313 (51.7%)		19 343 (46.5%)	86 197 (45.7%)	
LDL cholesterol (mmol/L)						
Mean (SD)	3.32 (0.8)	3.35 (0.8)	0.38	3.75 (0.8)	3.71 (0.8)	<0.001
Median [Min, Max]	3.29 [1.29, 5.90]	3.28 [0.85, 7.08]		3.71 [0.80, 7.84]	3.66 [0.27, 9.74]	
Smoking status						
Non‐smoker, *n* (%)	568 (81.1%)	2052 (80.8%)	0.89	26 892 (64.6%)	125 668 (66.7%)	<0.001
Smoker, *n* (%)	132 (18.9%)	487 (19.2%)		14 712 (35.4%)	62 843 (33.3%)	
Systolic blood pressure (mmHg)						
Mean (SD)	139 (21)	140 (21)	0.08	138 (20)	139 (20)	<0.001
Median [Min, Max]	136 [91, 236]	138 [98, 255]		136 [72, 253]	137 [80, 268]	
Diastolic blood pressure (mmHg)						
Mean (SD)	87 (13)	87 (12)	0.84	84 (11)	83 (11)	<0.001
Median [Min, Max]	86 [54, 126]	86 [54, 134]		84 [46, 144]	83 [42, 148]	
Takes blood pressure lowering medication						
No, *n* (%)	538 (76.9%)	1958 (77.1%)	0.93	36 132 (86.8%)	167 574 (88.9%)	<0.001
Yes, *n* (%)	162 (23.1%)	581 (22.9%)		5472 (13.2%)	20 937 (11.1%)	
Alcohol status						
Never, *n* (%)	133 (19.0%)	398 (15.7%)	0.11	1424 (3.4%)	5659 (3.0%)	<0.001
Previous, *n* (%)	34 (4.9%)	122 (4.8%)		1472 (3.5%)	5691 (3.0%)	
Current, *n* (%)	533 (76.1%)	2019 (79.5%)		38 708 (93.0%)	177 161 (94.0%)	
Daily fruit and vegetable intake						
Mean (SD)	7.5 (5.13)	9.3 (6.75)	<0.001	6.8 (3.96)	8.2 (4.56)	<0.001
Median [Min, Max]	6.0 [0, 44]	8.0 [0, 72]		6.0 [0, 80]	7.0 [0, 130]	
Oily fish intake						
Mean (SD)	1.8 (0.98)	2.0 (1.01)	<0.001	1.5 (0.88)	1.7 (0.92)	<0.001
Median [Min, Max]	2.0 [0, 5]	2.0 [0, 5]		1.0 [0, 5]	2.0 [0, 5]	
Meat intake						
Mean (SD)	8.1 (2.98)	8.0 (3.24)	0.18	8.0 (2.66)	7.8 (2.83)	<0.001
Median [Min, Max]	8 [0, 19]	8.0 [0, 25]		8.0 [0, 20]	8.0 [0, 25]	
BMI (kg/m^2^)						
Mean (SD)	29.7 (5.57)	29.2 (5.11)	0.03	28.0 (5.19)	26.6 (4.35)	<0.001
Median [Min, Max]	28.8 [18.90, 59.50]	28.5 [17.70, 68.10]		27.2 [13.60, 65]	26.0 [12.10, 66.20]	

Abbreviation: LDL, low‐density lipoprotein.

^a^ Systolic blood pressure >140 mmHg or diastolic blood pressure >90 mmHg.

* *p*‐value is provided for the difference in physical activity level. Statistical analysis were performed using the chi‐squared test for categorical variables and ANOVA for numerical variables.

Table 2 shows the effect of obesity genetic liability, as a continuous variable, on the odds of stage 2 hypertension within the whole sample and across categories of physical activity. For the whole sample, each unit increase in the standardized obesity genetic liabilities for BMI (fully adjusted OR = 1.05, 95% CI = 1.04–1.06), WHR (fully adjusted OR = 1.04, 95% CI = 1.03–1.04) and WC (fully adjusted OR = 1.04, 95% CI = 1.03–1.05) were associated with an increased odds of hypertension in the minimally and fully adjusted models (Table 2).

**TABLE 2:** Association between obesity genetic liability and hypertension in the European Ancestry sample.

Genetic liability	Whole sample (*n* = 230 115)				Low physical activity group (*n* = 41 604)			Moderate/high physical activity group (*n* = 188 511)		
	Odds ratio	95% CI	*p*‐Value for odds ratio	Interaction *p*‐value*	Odds ratio	95% CI	*p*‐Value for odds ratio	Odds ratio	95% CI	*p*‐Value for odds ratio
Unadjusted odds ratio										
BMI	1.04	0.84–0.85	<2 × 10^−16^	0.02	1.07	1.05–1.10	6.64 × 10^−13^	1.03	1.03–1.04	2.97 × 10^−13^
WHR	1.03	1.02–1.04	1.39 × 10^−13^	0.60	1.04	1.02–1.07	1.18 × 10^−5^	1.03	1.02–1.04	9.84 × 10^−10^
WC	1.04	1.03–1.05	3.36 × 10^−16^	0.09	1.05	1.03–1.08	2.81 × 10^−7^	1.03	1.02–1.04	4.38 × 10^−11^
Minimally adjusted odds ratio^a^										
BMI	1.04	1.04–1.06	<2 × 10^−16^	0.04	1.08	1.05–1.10	2.24 × 10^−12^	1.04	1.03–1.05	<2 × 10^−16^
WHR	1.04	1.03–1.04	2.97 × 10^−15^	0.31	1.05	1.03–1.07	6.03 × 10^−7^	1.03	1.02–1.04	2.04 × 10^−10^
WC	1.04	1.03–1.05	4.36 × 10^−15^	0.14	1.05	1.03–1.08	1.41 × 10^−6^	1.03	1.02–1.04	1.87 × 10^−10^
Adjusted odds ratio^b^										
BMI	1.05	1.04–1.06	<2 × 10^−16^	0.04	1.08	1.06–1.10	5.89 × 10^−13^	1.05	1.04–1.06	<2 × 10^−16^
WHR	1.04	1.03–1.04	3.82 × 10^−15^	0.38	1.05	1.03–1.07	1.23 × 10^−06^	1.03	1.02–1.04	1.59 × 10^−10^
WC	1.04	1.03–1.05	<2 × 10^−16^	0.15	1.06	1.03–1.08	4.12 × 10^−07^	1.04	1.03–1.05	4.11 × 10^−13^

*Note*: Odds ratios are given for the effect of each unit increase in standardized genetic liability on stage 2 hypertension.

Abbreviations: BMI, Body Mass Index; CI, Confidence interval; WC, Waist Circumference; WHR, Waist Hip Ratio.

^a^ Minimally adjusted for age and sex.

^b^ Adjusted for age, sex, smoking status, alcohol status, meat and fish intake, fruit and vegetable intake, and low‐density lipoprotein cholesterol.

* *p*‐value is provided for the interaction between adiposity genetic liability and physical activity on an additive scale. Two groups of physical activity [Low vs. Moderate/High] were included in the interaction model.

Within the low physical activity group, each unit increase in the standardized obesity genetic liabilities for BMI (fully adjusted OR = 1.08, 95% CI = 1.06–1.10), WHR (fully adjusted OR = 1.05, 95% CI = 1.03–1.07) and WC (fully adjusted OR = 1.06, 95% CI = 1.03–1.08) was also associated with increased odds of hypertension in unadjusted, minimally and fully adjusted models. Similar results was also observed in the models for the moderate/high physical activity subgroups; fully adjusted OR's were 1.05 (95% CI = 1.04–1.06) for BMI, 1.03 (95% CI = 1.02–1.04) for WHR and 1.04 (95% CI = 1.03–1.05) for WC (Table 2).

A statistically significant interaction effect was observed with physical activity attenuating the association between BMI genetic liability and hypertension across all models within the whole sample (Table 2). This interaction was not statistically significant for WHR or WC obesity genetic liability.

Compared to participants with a combination of moderate/high physical activity and low BMI genetic liability (Table 3), the odds of stage 2 hypertension were significantly increased in participants with a combination of low physical activity and high BMI obesity genetic liability. The largest odds of stage 2 hypertension was observed among participants with a combination of low physical activity and high BMI obesity genetic liability. A similar pattern was also observed in the WHR and WC genetic liabilities, with the largest odds of stage 2 hypertension observed among participants with low physical activity and high obesity (WHR and WC) genetic liability.

**TABLE 3:** Prevalence of stage 2 hypertension across different obesity genetic liability and physical activity groups in individuals of European ancestry.

Genetic liability categories		Physical activity level	Hypertensive (*n*)	Non‐hypertensive (*n*)	% Hypertensive	Unadjusted			Minimally adjusted^a^			Adjusted^b^		
						Odds ratio^c^	95% CI^d^	*p*‐Value for Odds ratio^*^	Odds ratio^c^	95% CI^d^	*p*‐value for Odds ratio^*^	Odds ratio^c^	95% CI^d^	*p*‐Value for odds ratio^*^
BMI	Low	Moderate/High	28 083	34 702	44.73	1 (reference)			1 (reference)			1 (reference)		
	Low	Low	6218	7699	44.68	1.00	0.96–1.04	0.92	1.05	1.01–1.10	7.67 × 10^−03^	1.03	0.99–1.07	0.14
	Moderate	Moderate/High	28 758	34 215	45.67	1.04	1.02–1.06	8.29 × 10^−04^	1.04	1.02–1.07	2.79 × 10^−04^	1.05	1.02–1.07	2.51 × 10^−04^
	Moderate	Low	6379	7353	46.45	1.07	1.03–1.11	2.34 × 10^−04^	1.14	1.09–1.18	1.11 × 10^−10^	1.11	1.07–1.16	1.48 × 10^−07^
	High	Moderate/High	29 356	33 397	46.78	1.09	1.06–1.11	3.00 × 10^−13^	1.10	1.08–1.13	5.70 × 10^−16^	1.10	1.08–1.13	<2 × 10^−16^
	High	Low	6746	7209	48.34	1.16	1.12–1.20	9.27 × 10^−15^	1.23	1.18–1.28	<2 × 10^−16^	1.20	1.16–1.25	<2 × 10^−16^
WHR	Low	Moderate/High	28 184	34 650	44.85	1 (reference)			1 (reference)			1 (reference)		
	Low	Low	6324	7582	45.48	1.03	0.99–1.06	0.18	1.07	1.03–1.11	4.57 × 10^−04^	1.05	1.01–1.09	0.02
	Moderate	Moderate/High	28 856	34 040	45.88	1.04	1.02–1.07	2.65 × 10^−04^	1.05	1.02–1.07	1.62 × 10^−04^	1.04	1.02–1.07	2.93 × 10^−04^
	Moderate	Low	6418	7359	46.58	1.07	1.03–1.11	2.20 × 10^−04^	1.13	1.09–1.18	2.63 × 10^−10^	1.10	1.06–1.15	6.43 × 10^−07^
	High	Moderate/High	29 157	33 624	46.44	1.07	1.04–1.09	1.62 × 10^−08^	1.07	1.05–1.10	1.28 × 10^−08^	1.07	1.05–1.10	1.71 × 10^−08^
	High	Low	6601	7320	47.42	1.11	1.07–1.15	3.91 × 10^−08^	1.18	1.14–1.23	<2 × 10^−16^	1.15	1.11–1.20	1.42 × 10^−12^
WC	Low	Moderate/High	28 325	34 702	44.94	1 (reference)			1 (reference)			1 (reference)		
	Low	Low	6170	7537	45.01	1.00	0.97–1.04	0.88	1.06	1.02–1.10	4.76 × 10^−03^	1.04	1.00–1.08	0.08
	Moderate	Moderate/High	28 561	34 178	45.52	1.02	1.00–1.05	0.04	1.02	1.00–1.04	0.10	1.02	1.00–1.05	0.05
	Moderate	Low	6484	7454	46.52	1.07	1.03–1.11	7 × 10^−04^	1.12	1.08–1.16	1.62 × 10^−08^	1.09	1.05–1.14	6.85 × 10^−06^
	High	Moderate/High	29 311	33 434	46.71	1.07	1.05–1.10	2.77 × 10^−10^	1.08	1.05–1.10	3.56 × 10^−10^	1.09	1.06–1.11	5.29 × 10^−12^
	High	Low	6689	7270	47.92	1.13	1.09–1.17	1.65 × 10^−10^	1.19	1.14–1.24	<2 × 10^−16^	1.17	1.13–1.22	1.68 × 10^−15^

*Note*: Odds ratios show the risk of prevalent stage 2 hypertension for participants belonging to each combination compared with the reference group (low genetic liability combined with moderate/high physical activity).

Abbreviations: BMI, Body Mass Index; WC, Waist Circumference; WHR, Waist Hip Ratio.

^a^ Adjusted for age and sex.

^b^ Adjusted for age, sex, smoking status, alcohol status, meat and fish intake, fruit and vegetable intake, and low‐density lipoprotein cholesterol.

c, d, *, Odds ratio, 95% confidence interval and p‐value for odds ratio, are provided for the joint effect of obesity genetic liability and physical activity on stage 2 hypertension. The values are derived from logistic regression models.

### 3.2 African ancestry results

Within the African ancestry sample, 3239 participants were included (Table 1; Table S4). There was no significant difference between the low and moderate/high physical activity sample in terms of prevalence of hypertension (*p* = 0.34). Body mass index in the low physical activity group (29.7 kg/m^2^; SD = 5.57) was higher than in the moderate/high physical activity group (29.2 kg/m^2^; SD = 5.11; *p* = 3.12 × 10^−2^).

None of the obesity genetic liabilities were significantly associated with hypertension within the whole sample or within any of the physical activity level groups (Table 4). Similar results were identified in the sensitivity analysis, where the odds of stage 2 hypertension in the African sample was examined using European ancestry‐derived SNPs (Table S5). To estimate if this was due to missing data and a smaller African sample size, a regression analysis was performed following the imputation of the missing variables (covariates). This did not change the outcomes, with none of the obesity genetic liabilities being associated with the odds of hypertension across different categories of physical activity (*p* > 0.05) (Table S6).

**TABLE 4:** Association between obesity genetic liability and hypertension in the African Ancestry sample.

Genetic liability	Whole sample (*n* = 3239)				Low physical activity group (*n* = 700)			Moderate/high physical activity group (*n* = 2539)		
	Odds ratio	95% CI	*p*‐Value for odds ratio	Interaction *p*‐value^*^	Odds ratio	95% CI	*p*‐Value for odds ratio	Odds ratio	95% CI	*p*‐Value for odds ratio
Unadjusted odds ratio										
BMI	1.05	0.97–1.14	0.21	NA	1.02	0.87–1.21	0.78	1.06	0.97–1.16	0.20
WHR	1.00	0.93–1.07	0.95	NA	0.93	0.80–1.08	0.34	1.02	0.94–1.10	0.68
WC	0.97	0.89–1.06	0.52	NA	0.95	0.79–1.14	0.60	0.98	0.89–1.08	0.66
Minimally adjusted odds ratio^a^										
BMI	1.06	0.98–1.15	0.17	NA	1.06	0.89–1.27	0.50	1.06	0.97–1.16	0.22
WHR	1.01	0.94–1.09	0.82	NA	0.94	0.80–1.09	0.40	1.03	0.95–1.12	0.47
WC	0.97	0.89–1.07	0.57	NA	0.92	0.76–1.12	0.43	0.99	0.89–1.10	0.81
Adjusted odds ratio^b^										
BMI	1.06	0.98–1.15	0.16	NA	1.06	0.89–1.27	0.50	1.06	0.97–1.16	0.22
WHR	1.01	0.94–1.09	0.80	NA	0.94	0.81–1.10	0.46	1.03	0.95–1.12	0.46
WC	0.97	0.88–1.06	0.49	NA	0.92	0.75–1.12	0.39	0.98	0.88–1.09	0.69

*Note*: Odds ratios are given for the effect of each unit increase in standardized genetic liability on prevalence of stage 2 hypertension.

Abbreviations: BMI, body mass index; CI, confidence interval; WC, waist circumference; WHR, waist–hip ratio.

^a^ Adjusted for age and sex.

^b^ Adjusted for age, sex, smoking status, alcohol status, meat and fish intake, fruit and vegetable intake, and low‐density lipoprotein cholesterol.

^*^ Refers to *p*‐value for interaction between physical activity and adiposity genetic liability.

## 4 DISCUSSION

The main findings of this study were that (1) high obesity genetic liability increases the odds of hypertension, and (2) physical activity could attenuate the increased likelihood of hypertension caused by high BMI genetic liability, in European ancestry individuals.

The combined effect of obesity and physical activity on the prevalence and risk of hypertension has been investigated in non‐genetic based studies. Jackson and colleagues^13^ reported that the risk of hypertension was 37% higher in healthy weight inactive Australian women compared with healthy weight highly active women. In a Chinese population, participants with obesity and low levels of self‐reported physical activity had a higher risk of hypertension compared with participants with normal weight and high physical activity levels.^43^ Stenehjem and colleagues^14^ reported a 50% higher risk in obese men with low physical activity levels compared to a combination of normal weight and high physical activity levels. However, these studies did not consider the life course effect of genetic predisposition to obesity in their analysis.^13^, ^14^, ^43^ The present study focused on obesity genetic liabilities, rather than phenotypes, which has the potential to identify the risk of hypertension early in the life course and enable investigation of the influence of physical activity in individuals with different genetic predispositions to adiposity. The findings in the current study imply that interventions targeting physical activity could be beneficial in reducing the odds of hypertension in individuals who are genetically predisposed to obesity. Among individuals who are not genetically predisposed to obesity, physical activity does not appear to be associated with the odds of hypertension, meaning interventions may be less effective in these populations. These findings have significant importance for informing population groups that may benefit most from physical activity with respect to obesity genetic liability and prevalence of hypertension.

In European ancestry participants, combinations of moderate or high obesity genetic liability and low physical activity were associated with an increased odds of hypertension compared with the reference group (low genetic liability and moderate/high physical activity). The effect of the different combinations of obesity genetic liability and low physical activity on hypertension were smaller in the current study compared with previous research that focused on the association of obesity phenotype and physical activity.^13^, ^14^, ^43^ The use of genetic liability in the preset study, as opposed to obesity phenotype, extends knowledge in the field of genomics by demonstrating the complex interplay between gene and environmental factors in determining susceptibility to hypertension. The findings from the present study could inform public health policy in the sense that they show how varied effects of physical activity can impact the relationship between genetics and hypertension. As higher physical activity levels appeared to have a greater protective effect in individuals with increased obesity (BMI) genetic risk, future research and public health policy could consider the hypertension‐related benefits of physical activity interventions targeting genetically high‐risk groups, rather than the whole population.

Genetic predisposition to obesity was not associated with hypertension among African ancestry participants. Previous studies in African ancestry populations have revealed no association between obesity genotype and hypertension.^17^ Shi and colleagues^17^ used European specific BMI genetic liability to investigate its association with hypertension in a small African sample (*n* = 369), and found no association. These results are unexpected as there is evidence that both obesity^5^ and low physical activity^44^ are associated with the prevalence of hypertension in African ancestry individuals. The lack of an association between African ancestry‐specific genetic liability and hypertension in the present study could be due to an insufficient sample size. The African ancestry subset of the UKB used in the primary (*n* = 3239) and sensitivity analysis following the imputation of missing data (*n* = 4249) was smaller compared with the European sample, meaning reduced power. In addition, the low number of SNPs identified within the African ancestry data could explain the lack of association, as estimating genetic liability with low SNP numbers can compromise prediction accuracy.^45^ Future studies with larger sample sizes are needed to assess the combined effects of obesity genetic liability and physical activity on the odds of hypertension among African ancestry individuals. There are also differences in fat distribution in African ancestry individuals compared with European ancestry individuals. For example, body fat mass is lower and lean muscle mass is higher in African ancestry individuals compared with European ancestry individuals when compared at the same BMI.^46^ These differences in body composition could affect the associations of obesity genetic liability with hypertension across the European and African ancestry samples. It was not possible to explore the influence of fat and lean muscle mass in the present study due to an insufficient African ancestry sample to undertake subgroup analyses according to these variables. Future larger studies should investigate the potential influence of muscle and fat mass distribution when investigating genetic and environmental factors linked to hypertension across different ancestry populations.

The present study has several strengths, including the large European ancestry sample size providing statistical power for physical activity subgroup analyses. The wide array of relevant covariates collected by the UKB enabled adjustments for potential confounding factors, thereby facilitating a more accurate estimation of the likelihood of hypertension. In addition, the analysis was performed in a sample of generally healthy men and women from the UK population,^47^ which enables generalization of the results and can inform public health programs for healthy populations. The novel approach to investigating the combined effect of obesity genetic liability and physical activity on the odds of prevalent hypertension advances the body of knowledge by informing potential preventive strategies according to individuals' genetic predisposition.

A potential limitation of the study is that African populations are both diverse^48^ and their genetic data relatively understudied in terms of genomics research. Thus, the ability to accurately assess genetic liability may be limited compared with European ancestry populations. Furthermore, the use of genetic liability provides a life course insight and typically explains small variation in outcomes.^49^ Another limitation is that physical activity was assessed via self‐report, which generally overestimates physical activity levels.^50^ Accelerometry data are available for only a small number of African ancestry individuals in the UKB (*n* = 394) who would be eligible for analysis in the present study. Further studies using device measures of physical activity could produce a more accurate estimate of the combined effect of physical activity and obesity genetic liability on hypertension prevalence.

## 5 CONCLUSION

The findings of this study demonstrate that increasing obesity genetic liability is associated with hypertension prevalence. Higher levels of physical activity attenuate the increased odds of stage 2 hypertension associated with obesity genetic liability in individuals of European ancestry. Physical activity interventions, may, therefore be important in individuals who have a genetic tendency to becoming overweight and obese. Obesity genetic liability and physical activity did not appear to be associated with hypertension within the African ancestry sample of the UKB. Further research with larger samples of African ancestry participants is needed to better understand the role of obesity genetic liability and physical activity in the context hypertension risk in this population group.

## AUTHOR CONTRIBUTIONS

Conceptualization, R.P.; Data curation, C.H.; Formal analysis, C.H.; Funding acquisition, R.P.; Investigation, C.H.; Methodology, C.H., R.P. and D.P.B.; Project administration, R.P.; Resources, R.P.; Supervision, R.P., A.I.B. and D.P.B; Writing—original draft, C.H.; Writing—review and editing, C.H., A.I.B., D.P.B. and R.P. All authors have read and agreed to the published version of the manuscript.

## FUNDING INFORMATION

R.P. holds a fellowship supported by Rutherford Fund from Medical Research Council (MR/R026505/1 and MR/R026505/2). UKB genotyping was supported by the British Heart Foundation (grant SP/13/2/30111) for Large‐scale comprehensive genotyping of UKB for cardiometabolic traits and diseases: UK CardioMetabolic Consortium.

## CONFLICT OF INTEREST STATEMENT

The authors declare no conflict of interest.

## INSTITUTIONAL REVIEW BOARD STATEMENT

The study was conducted in accordance with the Declaration of Helsinki and approved by the Institutional Review Board (or Ethics Committee) of Brunel University London, College of Health, Medicine and Life Sciences (27684‐LR‐Jan/2021–29 901‐1).

## INFORMED CONSENT

Informed consent was obtained from all subjects involved in the study.

## Supporting information


Appendix S1.

## Data Availability

Not applicable.

## References

[1] Unger T , Borghi C , Charchar F , et al. 2020 International Society of Hypertension Global Hypertension Practice Guidelines. Hypertension. 2020;75(6):1334‐1357. doi:10.1161/HYPERTENSIONAHA.120.15026 32370572

[2] Aggarwal R , Chiu N , Wadhera RK , et al. Racial/ethnic disparities in hypertension prevalence, awareness, treatment, and control in the United States, 2013 to 2018. Hypertension. 2021;78(6):1719‐1726. doi:10.1161/HYPERTENSIONAHA.121.17570 34365809 PMC10861176

[3] de Oliveira CM , Ulbrich AZ , Neves FS , et al. Association between anthropometric indicators of adiposity and hypertension in a Brazilian population: Baependi heart study. PLoS One. 2017;12(10):e0185225. doi:10.1371/journal.pone.0185225 29023455 PMC5638240

[4] George C , Goedecke JH , Crowther NJ , et al. The role of body fat and fat distribution in hypertension risk in urban black south African women. PLoS One. 2016;11(5):e0154894. doi:10.1371/JOURNAL.PONE.0154894 27171011 PMC4865112

[5] Pisa PT , Micklesfield LK , Kagura J , Ramsay M , Crowther NJ , Norris SA . Different adiposity indices and their association with blood pressure and hypertension in middle‐aged urban black south African men and women: findings from the AWI‐GEN south African Soweto site. BMC Public Health. 2018;18(1):524. doi:10.1186/S12889-018-5443-4 29673339 PMC5907712

[6] Pires NF , Coelho‐Júnior HJ , Gambassi BB , et al. Combined aerobic and resistance exercises evokes longer reductions on ambulatory blood pressure in resistant hypertension: a randomized crossover trial. Cardiovasc Ther. 2020;2020:1‐11. doi:10.1155/2020/8157858 PMC7416229 32821284

[7] Creber C , Cooper RS , Plange‐Rhule J , et al. Independent association of resting energy expenditure with blood pressure: confirmation in populations of the African diaspora. BMC Cardiovasc Disord. 2018;18(1):4. doi:10.1186/S12872-017-0737-5 29320983 PMC5763572

[8] Cvejkus RK , Miljkovic I , Barone Gibbs B , Zmuda JM , Wheeler VW , Kuipers AL . Association of physical activity with blood pressure in African ancestry men. Prev Med Rep. 2021;23:101458. doi:10.1016/J.PMEDR.2021.101458 34194964 PMC8227803

[9] Ogwumike OO , Adeniyi AF , Dosa BT , Sanya AO , Awolola KO . Physical activity and pattern of blood pressure in postmenopausal women with hypertension in Nigeria. Ethiop J Health Sci. 2014;24(2):153‐160. doi:10.4314/EJHS.V24I2.8 24795517 PMC4006210

[10] Giontella A , Lotta LA , Overton JD , et al. Causal effect of adiposity measures on blood pressure traits in 2 urban Swedish cohorts: a mendelian randomization study. J Am Heart Assoc. 2021;10(13):20405. doi:10.1161/JAHA.120.020405 PMC8403279 34120448

[11] Censin JC , Peters SAE , Bovijn J , et al. Causal relationships between obesity and the leading causes of death in women and men. PLoS Genet. 2019;15(10):e1008405. doi:10.1371/journal.pgen.1008405 31647808 PMC6812754

[12] Arija V , Villalobos F , Pedret R , et al. Physical activity, cardiovascular health, quality of life and blood pressure control in hypertensive subjects: randomized clinical trial. Health Qual Life Outcomes. 2018;16(1):184. doi:10.1186/S12955-018-1008-6 30217193 PMC6137925

[13] Jackson C , Herber‐Gast GC , Brown W . Joint effects of physical activity and BMI on risk of hypertension in women: a longitudinal study. J Obes. 2014;2014:1‐7. doi:10.1155/2014/271532 PMC3941781 24669312

[14] Stenehjem JS , Hjerkind KV , Nilsen TIL . Adiposity, physical activity, and risk of hypertension: prospective data from the population‐based HUNT study, Norway. J Hum Hypertens. 2018;32(4):278‐286. doi:10.1038/s41371-018-0042-5 29483587

[15] Evangelou E , Warren HR , Mosen‐Ansorena D , et al. Genetic analysis of over one million people identifies 535 new loci associated with blood pressure traits Europe PMC funders group. Nat Genet. 2018;50(10):1412‐1425. doi:10.1038/s41588-018-0205-x 30224653 PMC6284793

[16] Vaura F , Kauko A , Suvila K , et al. Polygenic risk scores predict hypertension onset and cardiovascular risk. Hypertension. 2021;77(4):1119‐1127. doi:10.1161/HYPERTENSIONAHA.120.16471 33611940 PMC8025831

[17] Shi M , Chen W , Sun X , et al. Association of Genome‐Wide Polygenic Risk Score for body mass index with cardiometabolic health from childhood through midlife. Circ Genom Precis Med. 2022;15(4):e003375. doi:10.1161/CIRCGEN.121.003375 35675159

[18] Moon JY , Wang T , Sofer T , et al. Objectively measured physical activity, sedentary behavior, and genetic predisposition to obesity in U.S. Hispanics/Latinos: results from the hispanic community health study/study of Latinos (HCHS/SOL). Diabetes. 2017;66(12):3001‐3012. doi:10.2337/DB17-0573/-/DC1 28986399 PMC5697950

[19] Johnson W , Ong KK , Elks CE , et al. Modification of genetic influences on adiposity between 36 and 63 years of age by physical activity and smoking in the 1946 British birth cohort study. Nutr Diabetes. 2014;4(9):e136. doi:10.1038/NUTD.2014.33 25198238 PMC4183974

[20] UK Biobank . UK Biobank: Protocol for a Large‐Scale Prospective Epidemiological Resource (AMENDMENT ONE FINAL). 2007.

[21] Bycroft C , Freeman C , Petkova D , et al. Genome‐wide genetic data on ~500,000 UK Biobank participants. *bioRxiv* . Published online July 20, 2017:166298. doi:10.1101/166298

[22] Marchini J , O'Connell J , Delaneau O , Sharp K , et al. UK Biobank Phasing and Imputation Documentation Contributors to UK Biobank Phasing and Imputation. https://www.jiscmail.ac.uk/cgi‐bin/webadmin?A0=UKB‐GENETICS. Published online 2015. Accessed October 22, 2022

[23] Welsh S , Peakman T , Sheard S , Almond R . Comparison of DNA quantification methodology used in the DNA extraction protocol for the UK Biobank cohort. BMC Genomics. 2017;18(1):26. doi:10.1186/S12864-016-3391-X 28056765 PMC5217214

[24] Elliott P , Peakman TC . The UK Biobank sample handling and storage protocol for the collection, processing and archiving of human blood and urine. Int J Epidemiol. 2008;37(2):234‐244. doi:10.1093/ije/dym276 18381398

[25] Pazoki R , Dehghan A , Evangelou E , et al. Genetic predisposition to high blood pressure and lifestyle factors. Circulation. 2018;137(7):653‐661. doi:10.1161/CIRCULATIONAHA.117.030898 29254930

[26] Tobin MD , Sheehan NA , Scurrah KJ , Burton PR . Adjusting for treatment effects in studies of quantitative traits: antihypertensive therapy and systolic blood pressure. Stat Med. 2005;24(19):2911‐2935. doi:10.1002/SIM.2165 16152135

[27] Whelton PK , Carey RM , Aronow WS , et al. 2017 ACC/AHA/AAPA/ABC/ACPM/AGS/APhA/ASH/ASPC/NMA/PCNA guideline for the prevention, detection, evaluation, and Management of High Blood Pressure in adults: executive summary: a report of the American College of Cardiology/American Heart Association task force on clinical practice guidelines. J Am Coll Cardiol. 2018;71(19):2199‐2269. doi:10.1016/j.jacc.2017.11.005 29146535

[28] Craig CL , Marshall AL , Sjöström M , et al. International physical activity questionnaire: 12‐country reliability and validity. Med Sci Sports Exerc. 2003;35(8):1381‐1395. doi:10.1249/01.MSS.0000078924.61453.FB 12900694

[29] Cassidy S , Chau JY , Catt M , Bauman A , Trenell MI . Cross‐sectional study of diet, physical activity, television viewing and sleep duration in 233 110 adults from the UK Biobank; the behavioural phenotype of cardiovascular disease and type 2 diabetes. BMJ Open. 2016;6(3):e010038. doi:10.1136/bmjopen-2015-010038 PMC4800116 27008686

[30] IPAQ . Guidelines for Data Processing and Analysis of the International Physical Activity Questionnaire (IPAQ)‐Short and Long Forms. 2005 www.ipaq.ki.se

[31] World Health Organisation . WHO GUIDELINES ON PHYSICAL ACTIVITY AND SEDENTARY BEHAVIOUR. Published online 2020. Accessed March 14, 2024. https://iris.who.int/bitstream/handle/10665/336656/9789240015128‐eng.pdf?sequence=1

[32] UK Biobank . Showcase: data‐field 20161. 2016 Accessed June 29, 2023. https://biobank.ndph.ox.ac.uk/showcase/field.cgi?id=20161

[33] UK Biobank . UK Biobank Biomarker assay quality procedures: approaches used to minimise systematic and random errors (and the wider epidemiological implications). http://www.ukbiobank.ac.uk/. Published online 2019. Accessed October 22, 2022. http://www.ukbiobank.ac.uk/

[34] Winkler TW , Justice AE , Graff M , et al. The influence of age and sex on genetic associations with adult body size and shape: a large‐scale genome‐wide interaction study. PLoS Genet. 2015;11(10):e1005378. doi:10.1371/JOURNAL.PGEN.1005378 26426971 PMC4591371

[35] Ng MCY , Graff M , Lu Y , et al. Discovery and fine‐mapping of adiposity loci using high density imputation of genome‐wide association studies in individuals of African ancestry: African ancestry anthropometry genetics consortium. PLoS Genet. 2017;13(4):81. doi:10.1371/JOURNAL.PGEN.1006719 PMC5419579 28430825

[36] Shungin D , Winkler T , Croteau‐Chonka DC , et al. New genetic loci link adipose and insulin biology to body fat distribution. Nature. 2015;518(7538):187‐196. doi:10.1038/nature14132 25673412 PMC4338562

[37] Liu CT , Monda KL , Taylor KC , et al. Genome‐wide Association of Body fat Distribution in African ancestry populations suggests new loci. PLoS Genet. 2013;9(8):e1003681. doi:10.1371/JOURNAL.PGEN.1003681 23966867 PMC3744443

[38] Purcell S , Neale B , Todd‐Brown K , et al. PLINK: a tool set for whole‐genome association and population‐based linkage analyses. Am J Hum Genet. 2007;81(3):559‐575. doi:10.1086/519795 17701901 PMC1950838

[39] MacQueen J . Some methods for classification and analysis of multivariate observations. Fifth Berkeley Symposium on Mathematical Statistics and Probability. University of California Press; 1967:281‐297.

[40] Henry D , Dymnicki AB , Mohatt N , Allen J , Kelly JG . Clustering methods with qualitative data: a mixed methods approach for prevention research with small samples. Prev Sci. 2015;16(7):1007‐1016. doi:10.1007/S11121-015-0561-Z 25946969 PMC4939904

[41] Van Buuren S , Groothuis‐Oudshoorn K . mice: Multivariate imputation by chained equations in R. J Stat Softw. 2011;45(3):1‐67.

[42] R Core Team . R: The R Project for Statistical Computing. Published 2021. Accessed September 23, 2021. https://www.r‐project.org/

[43] Li W , Wang D , Wu C , Shi O , Zhou Y , Lu Z . The effect of body mass index and physical activity on hypertension among Chinese middle‐aged and older population. Sci Rep. 2017;7(1):10256. doi:10.1038/S41598-017-11037-Y 28860562 PMC5579023

[44] Diaz KM , Booth JN , Seals SR , et al. Physical activity and incident hypertension in African Americans. Hypertension. 2017;69(3):421‐427. doi:10.1161/HYPERTENSIONAHA.116.08398 28137988 PMC5302780

[45] Chagnon M , O'Loughlin J , Engert JC , Karp I , Sylvestre MP . Missing single nucleotide polymorphisms in genetic risk scores: a simulation study. PLoS One. 2018;13(7):e0200630. doi:10.1371/JOURNAL.PONE.0200630 30024900 PMC6053141

[46] Rush EC , Goedecke JH , Jennings C , et al. BMI, fat and muscle differences in urban women of five ethnicities from two countries. Int J Obes. 2007;31(8):1232‐1239. doi:10.1038/sj.ijo.0803576 17342075

[47] Fry A , Littlejohns TJ , Sudlow C , et al. Comparison of sociodemographic and health‐related characteristics of UK Biobank participants with those of the general population. Am J Epidemiol. 2017;186(9):1026‐1034. doi:10.1093/AJE/KWX246 28641372 PMC5860371

[48] Retshabile G , Mlotshwa BC , Williams L , et al. Whole‐exome sequencing reveals uncaptured variation and distinct ancestry in the southern African population of Botswana. Am J Hum Genet. 2018;102(5):731‐743. doi:10.1016/j.ajhg.2018.03.010 29706352 PMC5986695

[49] Narusyte J , Ropponen A , Silventoinen K , et al. Genetic liability to disability pension in women and men: a prospective population‐based twin study. PLoS One. 2011;6(8):e23143. doi:10.1371/journal.pone.0023143 21850258 PMC3151284

[50] Luo J , Lee RYW . Opposing patterns in self‐reported and measured physical activity levels in middle‐aged adults. Eur J Ageing. 2022;19(3):567‐573. doi:10.1007/S10433-021-00657-Z/TABLES/6 36052191 PMC9424380

