# Supplementary material for: Physical activity alters the effect of genetic determinants of adiposity on hypertension among individuals of European ancestry in the UKB
Source: Scand J Med Sci Sports. 2024 Apr 26;34(5):e14636. doi: 10.1111/sms.14636 (PMC11135603; doi:10.1111/sms.14636)
Supplement: Supplementary file 1 — Appendix S1. [file SMS-34-e14636-s001.docx]

**Supplementary Material for**

**Physical activity alters the effect of genetic determinants of adiposity on hypertension among individuals of European ancestry in the UKB**

Chukwueloka HEZEKIAH, MSc, Alexandra I BLAKEMORE, PhD, Daniel P BAILEY, PhD and Raha PAZOKI, MD, PhD


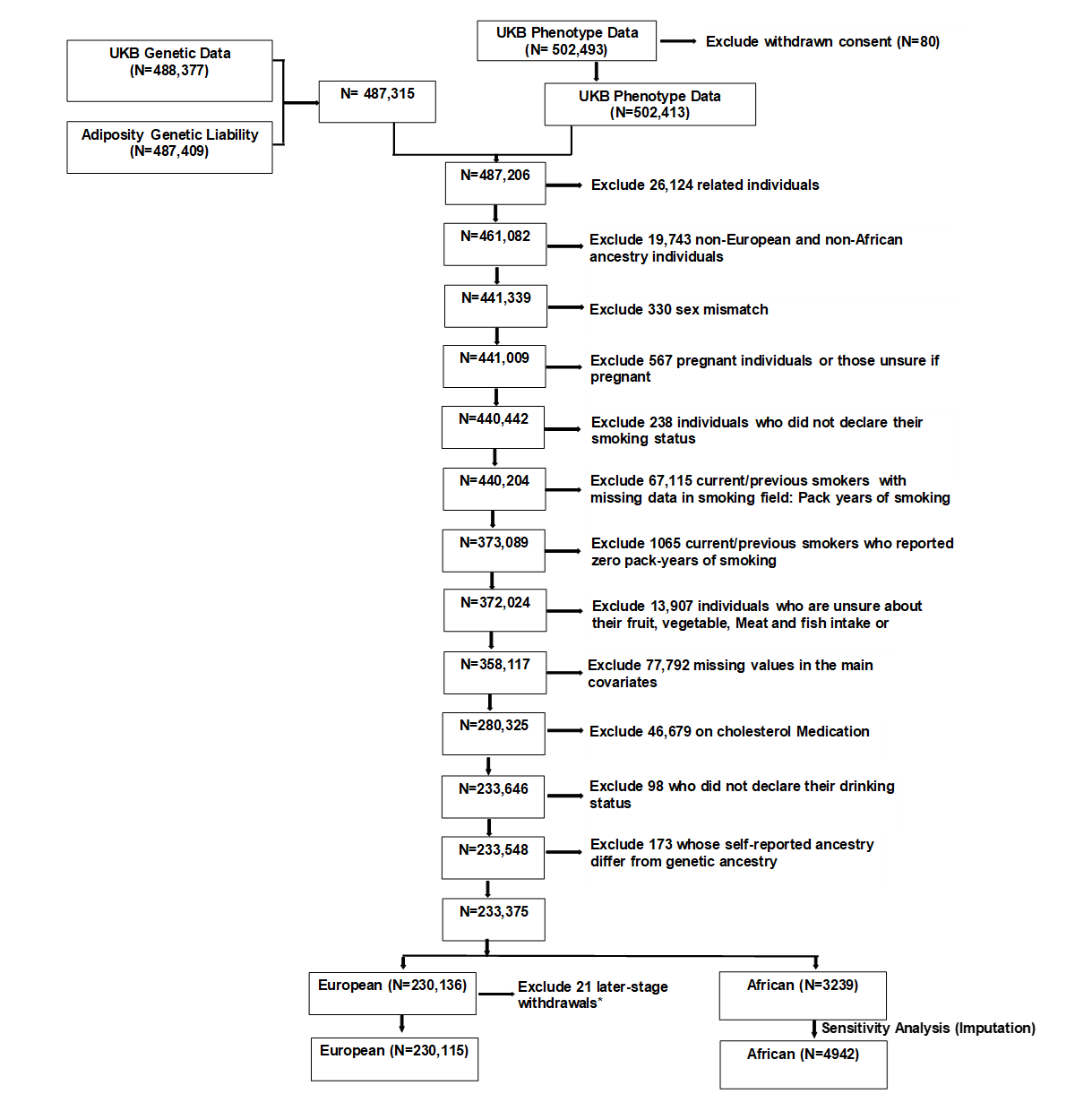


Supplementary Figure 1. Flow chart of eligible population illustrating the phenotype and genotype quality control within the UK Biobank data.

* Participants who withdrew their consent after the analysis was completed.

Supplementary Table 1. List of SNPs used for the generation of obesity genetic liability for European ancestry following pruning.

| EUROPEAN BMI | | | EUROPEAN BMI continued. | | | EUROPEAN BMI continued. | | |
| --- | --- | --- | --- | --- | --- | --- | --- | --- |
| SNP | Effect Allele | Effect Estimate | SNP | Effect Allele | Effect Estimate | SNP | Effect Allele | Effect Estimate |
| rs11583200 | T | -0.015170207 | rs11727676 | T | 0.0379 | rs10840100 | A | -0.0222 |
| rs990871 | T | 0.033314496 | rs1503526 | T | -0.0155 | rs16917237 | T | -0.0409 |
| rs7553348 | A | -0.022879148 | rs6870983 | T | -0.0198 | rs4755726 | T | 0.0169 |
| rs17381664 | T | -0.021559669 | rs7713317 | A | -0.0178 | rs7124681 | A | 0.0245 |
| rs11165643 | T | 0.023304291 | rs158186 | A | -0.0263 | rs12286929 | A | -0.0181 |
| rs7550711 | T | 0.06373383 | rs6864049 | A | -0.0182 | rs7928810 | A | 0.0159 |
| rs543874 | A | -0.048511162 | rs13174863 | A | -0.0195 | rs1816537 | A | -0.0190 |
| rs2803328 | C | -0.016034162 | rs2112347 | T | 0.0238 | rs7138803 | A | 0.0327 |
| rs7535528 | A | -0.018895956 | rs815610 | C | 0.0205 | rs10846664 | A | -0.0176 |
| rs2271928 | A | -0.017041538 | rs205262 | A | -0.0232 | rs17630235 | A | -0.0194 |
| rs2275426 | A | 0.017272513 | rs2206277 | T | 0.0413 | rs729062 | A | -0.0378 |
| rs4512652 | C | -0.019193487 | rs9400239 | T | -0.0171 | rs11247009 | A | 0.0248 |
| rs10913118 | A | -0.015760293 | rs9374842 | T | 0.0164 | rs11611246 | T | 0.0242 |
| rs10920678 | A | 0.016999872 | rs13191362 | A | 0.0273 | rs7970953 | A | 0.0167 |
| rs977747 | T | 0.016 | rs2228213 | A | -0.0167 | rs1405552 | A | -0.0175 |
| rs2820292 | A | -0.01753016 | rs943466 | A | -0.0183 | rs10777237 | T | 0.0213 |
| rs492400 | T | -0.013458029 | rs13201877 | A | -0.0208 | rs12016871 | T | 0.0277 |
| rs972540 | A | -0.018581709 | rs1167827 | A | -0.0204 | rs12429545 | A | 0.0339 |
| rs7421089 | T | 0.019202283 | rs17149254 | T | 0.0375 | rs9540493 | A | 0.0173 |
| rs3739081 | A | 0.015953999 | rs9641123 | C | 0.0172 | rs2504236 | T | -0.0169 |
| rs13011109 | C | -0.021146797 | rs6465468 | T | 0.0170 | rs9634489 | A | -0.0176 |
| rs1016287 | T | 0.021626975 | rs1830074 | T | -0.0192 | rs1441264 | A | 0.0152 |
| rs11688816 | A | -0.014876516 | rs17161076 | A | -0.0201 | rs10132280 | A | -0.0213 |
| rs2890652 | T | -0.027040575 | rs11765748 | A | -0.0186 | rs12885454 | A | -0.0213 |
| rs1460678 | C | -0.025796715 | rs4727804 | A | -0.0208 | rs11847697 | T | 0.0469 |
| rs7588437 | A | -0.019029135 | rs17405819 | T | 0.0235 | rs7141420 | T | 0.0275 |
| rs11692326 | T | 0.018731855 | rs16907751 | T | -0.0374 | rs17522122 | T | 0.0163 |
| rs4072096 | A | -0.016502118 | rs733594 | T | 0.0173 | rs3783890 | T | 0.0189 |
| rs1840969 | A | -0.019002179 | rs6990042 | T | -0.0161 | rs7143963 | T | 0.0205 |
| rs876424 | T | -0.018912453 | rs10954968 | A | 0.0188 | rs709400 | A | 0.0164 |
| rs2649734 | A | -0.016696025 | rs6994670 | A | 0.0535 | rs3736485 | A | 0.0171 |
| rs9845966 | T | 0.015592072 | rs3134353 | A | -0.0175 | rs2241423 | A | -0.0327 |
| rs7613875 | A | 0.015881946 | rs4740619 | T | 0.0171 | rs8027181 | A | -0.0193 |
| rs10510554 | T | -0.017831419 | rs10968577 | T | 0.0250 | rs12593036 | A | 0.0183 |
| rs2365389 | T | -0.019783955 | rs6477694 | T | -0.0194 | rs7181659 | A | 0.0147 |
| rs6792696 | A | 0.018291831 | rs1928295 | T | 0.0191 | rs9925964 | A | 0.0199 |
| rs13068138 | T | 0.027749745 | rs10733682 | A | 0.0184 | rs758747 | T | 0.0172 |
| rs16851483 | T | 0.048569173 | rs10760279 | T | 0.0216 | rs2531995 | T | 0.0249 |
| rs1516725 | T | -0.04735101 | rs2270204 | T | -0.0191 | rs12446632 | A | -0.0426 |
| rs16992647 | T | -0.02042858 | rs7899106 | A | -0.0452 | rs2650492 | A | 0.0243 |
| rs4833407 | A | 0.016669314 | rs17094222 | T | -0.0205 | rs7204797 | T | 0.0183 |
| rs10938397 | A | -0.040631566 | rs11191514 | T | 0.0313 | rs1000940 | A | -0.0175 |
| rs17001654 | C | -0.029506063 | rs7903146 | T | -0.0219 | rs12939549 | A | 0.0175 |
| rs13107325 | T | 0.0492 | rs10886017 | A | 0.0172 | rs12150665 | T | 0.0197 |
| rs11652097 | T | 0.0155 | rs6567160 | T | -0.0538 | rs3810291 | A | 0.0279 |
| rs6504108 | T | -0.0176 | rs7231852 | A | -0.0187 | rs17513613 | T | -0.0171 |
| rs17631394 | A | -0.0226 | rs7239883 | A | -0.0143 | rs33439 | T | 0.0174 |
| rs312750 | A | 0.0148 | rs17724992 | A | 0.0173 | rs6091540 | T | -0.0185 |
| rs9914578 | C | -0.0179 | rs29938 | T | -0.0216 | rs175804 | A | 0.0247 |
| rs6507716 | A | 0.0194 | rs2075650 | A | 0.0257 | rs2836754 | T | -0.0129 |
| rs7243357 | T | 0.0231 | rs11672660 | T | -0.0289 | rs4820408 | T | 0.0156 |
| rs11866815 | T | -0.0208 | rs889398 | T | -0.0178 |  |  |  |

Supplementary Table 1 (continued). List of SNPs used for the generation of obesity genetic liability for European ancestry following pruning.

| EUROPEAN WHR | | | EUROPEAN WC | | |
| --- | --- | --- | --- | --- | --- |
| SNP | Effect Allele | Effect Estimate | SNP | Effect Allele | Effect Estimate |
| rs2765539 | T | 0.0271 | rs633715 | T | -0.0431 |
| rs1011731 | A | -0.0191 | rs11165623 | A | 0.0198 |
| rs4846565 | A | -0.0231 | rs7550711 | T | 0.0577 |
| rs10195252 | T | 0.0199 | rs3127553 | A | -0.0226 |
| rs929641 | A | 0.0197 | rs4130548 | T | -0.022 |
| rs17819328 | T | -0.0163 | rs2820292 | A | -0.0189 |
| rs10804591 | A | 0.0214 | rs7531118 | T | -0.0268 |
| rs17451107 | T | 0.0232 | rs6755502 | T | -0.0512 |
| rs9860730 | A | 0.0229 | rs929641 | A | 0.0207 |
| rs9491696 | C | -0.0379 | rs6545714 | A | -0.022 |
| rs1358980 | T | 0.027 | rs3849570 | A | 0.0214 |
| rs4715208 | A | -0.0186 | rs6440003 | A | 0.0215 |
| rs1294421 | T | -0.0255 | rs2325036 | A | 0.0232 |
| rs7801581 | T | 0.0225 | rs1516725 | T | -0.031 |
| rs12549058 | T | -0.0402 | rs10938397 | A | -0.0316 |
| rs4929927 | A | -0.0198 | rs2112347 | T | 0.0254 |
| rs863750 | T | 0.0163 | rs806794 | A | 0.0223 |
| rs11048470 | T | 0.0253 | rs943005 | T | 0.0388 |
| rs1440372 | T | -0.0213 | rs9400239 | T | -0.0244 |
| rs1121980 | A | 0.0432 | rs2489623 | A | -0.0187 |
| rs4646404 | A | -0.0196 | rs16894959 | T | -0.0262 |
| rs4640244 | A | -0.0207 | rs10968576 | A | -0.0246 |
| rs11663816 | T | -0.025 | rs7903146 | T | -0.0219 |
| rs2075650 | A | 0.0286 | rs6163 | A | 0.0191 |
| rs2287019 | T | -0.0263 | rs10767658 | C | 0.0312 |
| rs16996700 | T | 0.0209 | rs2293576 | A | -0.0222 |
| rs2179129 | A | 0.0207 | rs10840100 | A | -0.0203 |
|  |  |  | rs7138803 | A | 0.0282 |
|  |  |  | rs7144011 | T | 0.033 |
|  |  |  | rs4776970 | A | 0.0196 |
|  |  |  | rs2650492 | A | 0.0256 |
|  |  |  | rs1558902 | A | 0.0739 |
|  |  |  | rs1549293 | T | -0.0201 |
|  |  |  | rs12446632 | A | -0.0361 |
|  |  |  | rs2531992 | A | -0.0282 |
|  |  |  | rs6567160 | T | -0.0483 |
|  |  |  | rs7239883 | A | -0.0207 |
|  |  |  | rs3810291 | A | 0.0259 |
|  |  |  | rs2075650 | A | 0.0307 |
|  |  |  | rs2287019 | T | -0.0351 |
|  |  |  | rs16996700 | T | 0.0226 |

All known SNPs from previously reported genetic variants for BMI ^1^, WHR ^2^ and WC ^2^ and their effect size have been included. SNPs are pairwise independent and not in linkage disequilibrium. BMI: Body Mass Index, WHR: Waist Hip Ratio, WC: Waist Circumference, SNP: Single Nucleotide Polymorphism.

Supplementary Table 2. List of SNPs used for the generation of obesity genetic liability for African ancestry following pruning.

| AFRICAN BMI | | | AFRICAN WHR | | | AFRICAN WC | | |
| --- | --- | --- | --- | --- | --- | --- | --- | --- |
| SNP | Effect Allele | Effect Estimate | SNP | Effect Allele | Effect Estimate | SNP | Effect Allele | Effect Estimate |
| rs543874 | G | 0.055 | rs6931262 | T | 0.06 | rs2075064 | T | -0.07 |
| rs62105306 | T | 0.053 | rs1294410 | T | -0.05 | rs7601155 | T | 0.06 |
| rs10938397 | G | 0.044 | rs10894604 | T | -0.06 |  |  |  |
| rs7708584 | A | 0.054 |  |  |  |  |  |  |
| rs17057164 | T | 0.04 |  |  |  |  |  |  |
| rs17817964 | T | 0.069 |  |  |  |  |  |  |
| rs6567160 | C | 0.059 |  |  |  |  |  |  |

BMI: Body Mass Index, WHR: Waist Hip Ratio, WC: Waist Circumference, SNP: Single Nucleotide Polymorphism. All known SNPs from previously reported genetic variants for BMI ^3^, WHR ^4^ and WC ^4^ and their effect size have been included. SNPs are pairwise independent and not in linkage disequilibrium.

Supplementary Table 3. The relationship between the UK Biobank self-reported ancestry with genetic based clusters following K-means clustering analysis (n=488,247).

| **Genetically Derived Clusters** | **UKB Self-Reported Ancestry** | | | | | | |
| --- | --- | --- | --- | --- | --- | --- | --- |
|  | **African** | **Asian** | **Chinese** | **Missing** | **Mixed** | **Other** | **White** |
| **1** | 85 | 8750 | 1 | 253 | 385 | 583 | 71 |
| **2** | 11 | 19 | 3 | 357 | 259 | 66 | 131,138 |
| **3** | 191 | 275 | 1 | 416 | 469 | 1476 | 96,629 |
| **4** | 31 | 48 | 1 | 751 | 794 | 528 | 180,077 |
| **5** | 7 | 11 | 1 | 225 | 190 | 250 | 52,166 |
| **6** | 7316 | 1 | 0 | 228 | 554 | 800 | 5 |
| **7** | 4 | 370 | 1497 | 78 | 192 | 653 | 31 |

Supplementary Table 4. Baseline characteristics of the study sample by sex

| Characteristics | African | | | European | | |
| --- | --- | --- | --- | --- | --- | --- |
|  | Female  n=1959 | Male  n=1280 | *P*-value for sex difference* | Female  n=128,277 | Male  n=101,838 | P-value for sex difference * |
| Age (Years) |  |  |  |  |  |  |
| Mean (SD)  Median [Min, Max] | 50.9 (7.3)  50.0 [40, 70] | 50.3 (7.7)  49.0 [40, 70] | 0.04 | 55.4 (7.9)  56.0 [40, 70] | 55.4 (8.1)  56.0 [38, 73] | 0.04 |
| Stage 2 hypertension^†^ |  |  |  |  |  |  |
| No, n (%)  Yes, n (%) | 962 (49.1%)  997 (50.9%) | 617 (48.2%)  663 (51.8%) | 0.64 | 76,612 (59.7%)  51,665 (40.3%) | 47,963 (47.1%)  53,875 (52.9%) | <0.001 |
| Physical Activity Level |  |  |  |  |  |  |
| Low, n (%)  Moderate/High, n (%) | 419 (21.4%)  1540 (78.6%) | 281 (22.0%)  999 (78.0%) | 0.74 | 23,099 (18.0%)  105,178 (82.0%) | 18,505 (18.2%)  83,333 (81.8%) | 0.31 |
| LDL Cholesterol (mmol/L) |  |  |  |  |  |  |
| Mean (SD)  Median [Min, Max] | 3.32 (0.8)  3.27 [0.85, 7.08] | 3.38 (0.8)  3.32 [1.29, 7.06] | 0.07 | 3.72 (0.8)  3.67 [0.80, 9.74] | 3.70 (0.8)  3.67 [0.27, 8.99] | <0.001 |
| Smoking Status |  |  |  |  |  |  |
| Non-Smoker, n (%)  Smoker, n (%) | 1650 (84.2%)  309 (15.8%) | 970 (75.8%)  310 (24.2%) | <0.001 | 88,922 (69.3%)  39,355 (30.7%) | 63,638 (62.5%)  38,200 (37.5%) | <0.001 |
| Systolic Blood Pressure (mmHg) |  |  |  |  |  |  |
| Mean (SD)  Median [Min, Max] | 139 (22)  136 [91, 255] | 142 (19)  139 [103, 236] | <0.001 | 136 (21  133 [72, 253] | 142 (19)  140 [85, 268] | <0.001 |
| Diastolic Blood Pressure (mmHg) |  |  |  |  |  |  |
| Mean (SD)  Median [Min, Max] | 87 (12)  86 [53.50, 134] | 87 (12)  86 [55, 129] | 0.63 | 82 (11)  80.50 [45, 142] | 86 (11)  85 [42, 148] | <0.001 |
| Takes Blood Pressure Lowering Medication |  |  |  |  |  |  |
| No, n (%)  Yes, n (%) | 1466 (74.8%)  493 (25.2%) | 1030 (80.5%)  250 (19.5%) | <0.001 | 114,369 (89.2%)  13,908 (10.8%) | 89,337 (87.7%)  12,501 (12.3%) | <0.001 |
| Alcohol Status |  |  |  |  |  |  |
| Never, n (%)  Previous, n (%)  Current, n (%) | 355 (18.1%)  80 (4.1%)  1524 (77.8%) | 176 (13.8%)  76 (5.9%)  1028 (80.3%) | <0.001 | 5272 (4.1%)  4161 (3.2%)  118,844 (92.6%) | 1811 (1.8%)  3002 (2.9%)  97,025 (95.3%) | <0.001 |
| Daily Fruit and Vegetable Intake |  |  |  |  |  |  |
| Mean (SD)  Median [Min, Max] | 9.2 (5.78)  8 [0, 53] | 8.6 (7.40)  7 [0, 72] | 0.02 | 8.4 (4.39)  8 [0, 106] | 7.3 (4.54)  6.50 [0, 13] | <0.001 |
| Oily Fish Intake |  |  |  |  |  |  |
| Mean (SD)  Median [Min, Max] | 2.0 (0.99)  2 [0, 5] | 1.9 (1.01)  2 [0, 5] | 0.05 | 1.7 (0.91)  2 [0, 5] | 1.6 (0.92)  2 [0, 5] | <0.001 |
| Meat Intake |  |  |  |  |  |  |
| Mean (SD)  Median [Min, Max] | 7.4 (3.04)  7 [0, 23] | 8.9 (3.20)  9 [0, 25] | <0.001 | 7.3 (2.81)  8 [0, 25] | 8.4 (2.66)  9 [0, 25] | <0.001 |
| BMI (kg/m^2^) |  |  |  |  |  |  |
| Mean (SD)  Median [Min, Max] | 29.9 (5.71)  29.2 [18.20, 68.10] | 28.3 (4.18)  27.9 [17.70, 57.5] | <0.001 | 26.5 (4.88)  25.6 [12.10, 66.20] | 27.3 (4.03)  26.9 [12.80, 61.70] | <0.001 |

* P-value is included for sex difference. Statistical analyses were performed using the chi-squared test, for categorical variables and ANOVA, for numerical variables. † Systolic blood pressure >140 or diastolic blood pressure >90. LDL, low-density lipoprotein.

Supplementary Table 5. Association between obesity genetic liability and hypertension in African Ancestry sample using European ancestry-derived genetic liability.

Odds ratios are given for the effect of each unit increase in standardised genetic liability on stage 2 hypertension. ^a^ Adjusted for age and sex. ^b^ Adjusted for age, sex, smoking status, alcohol status, meat and fish intake, fruit and vegetable intake, and low-density lipoprotein cholesterol. BMI, Body Mass Index; WHR, Waist Hip Ratio; WC, Waist Circumference; CI, confidence interval.

| Genetic Liability | Whole Sample  (n=3239) | | | | Low Physical Activity Group  (n=700) | | | Moderate/High Physical Activity Group  (n=2539) | | |
| --- | --- | --- | --- | --- | --- | --- | --- | --- | --- | --- |
|  | Odds Ratio | 95% CI | *P*-value for Odds Ratio | Interaction *P*-value * | Odds Ratio | 95% CI | *P*-value for Odds Ratio | Odds Ratio | 95% CI | *P*-value for Odds Ratio |
| Unadjusted Odds Ratio | | | | | | | | | | |
| BMI | 1.00 | 0.92 - 1.08 | 0.93 | NA | 1.06 | 0.89 - 1.28 | 0.51 | 1.00 | 0.91 - 1.09 | 0.92 |
| WHR | 1.00 | 0.92 - 1.08 | 0.96 | NA | 0.93 | 0.78 - 1.10 | 0.38 | 1.02 | 0.93 - 1.11 | 0.70 |
| WC | 0.91 | 0.84 - 0.99 | 0.03 | 0.25 | 0.81 | 0.67 - 0.97 | 0.03 | 0.94 | 0.85 - 1.03 | 0.18 |
| Minimally Adjusted Odds Ratio ^a^ | | | | | | | | | | |
| BMI | 0.99 | 0.91 - 1.08 | 0.87 | NA | 1.06 | 0.89 - 1.28 | 0.51 | 0.98 | 0.89 - 1.07 | 0.62 |
| WHR | 1.01 | 0.93 - 1.09 | 0.87 | NA | 0.91 | 0.76 - 1.09 | 0.28 | 1.03 | 0.94 - 1.13 | 0.48 |
| WC | 0.93 | 0.85 - 1.02 | 0.11 | NA | 0.89 | 0.73 - 1.09 | 0.28 | 0.94 | 0.85 - 1.04 | 0.23 |
| Adjusted Odds Ratio ^b^ | | | | | | | | | | |
| BMI | 1.00 | 0.92 - 1.08 | 0.96 | NA | 1.07 | 0.89 - 1.29 | 0.46 | 0.98 | 0.89 - 1.08 | 0.71 |
| WHR | 1.01 | 0.93 - 1.09 | 0.84 | NA | 0.90 | 0.75 - 1.09 | 0.28 | 1.04 | 0.95 - 1.14 | 0.43 |
| WC | 0.93 | 0.85 - 1.02 | 0.13 | NA | 0.89 | 0.78 - 1.09 | 0.27 | 0.95 | 0.86 - 1.05 | 0.29 |

Supplementary Table 6. Association between obesity genetic liability and hypertension in the African Ancestry sample using the imputed dataset.

| Genetic Liability | Whole Sample  (n=4942) | | | | Low Physical Activity Group  (n=1060) | | | Moderate/High Physical Activity Group  (n=3882) | | |
| --- | --- | --- | --- | --- | --- | --- | --- | --- | --- | --- |
|  | Odds Ratio | 95% CI | *P*-value for Odds Ratio | Interaction *P*-value * | Odds Ratio | 95% CI | *P*-value for Odds Ratio | Odds Ratio | 95% CI | *P*-value for Odds Ratio |
| Unadjusted Odds Ratio | | | | | | | | | | |
| BMI | 1.03 | 0.97 - 1.10 | 0.36 | N/A | 0.97 | 0.85 - 1.11 | 0.63 | 1.05 | 0.98 - 1.13 | 0.20 |
| WHR | 1.01 | 0.95 - 1.07 | 0.78 | N/A | 0.95 | 0.84 - 1.07 | 0.41 | 1.02 | 0.96 - 1.09 | 0.47 |
| WC | 1.01 | 0.94 - 1.08 | 0.89 | N/A | 0.99 | 0.85 - 1.16 | 0.94 | 1.01 | 0.93 - 1.09 | 0.84 |
| Minimally Adjusted Odds Ratio ^a^ | | | | | | | | | | |
| BMI | 1.03 | 0.96 - 1.10 | 0.42 | N/A | 0.97 | 0.84 - 1.12 | 0.65 | 1.05 | 0.97 - 1.13 | 0.24 |
| WHR | 1.02 | 0.96 - 1.08 | 0.60 | N/A | 0.96 | 0.84 - 1.09 | 0.53 | 1.03 | 0.97 - 1.11 | 0.34 |
| WC | 1.01 | 0.94 - 1.09 | 0.81 | N/A | 0.97 | 0.83 - 1.14 | 0.73 | 1.02 | 0.94 - 1.11 | 0.66 |
| Adjusted Odds Ratio ^b^ | | | | | | | | | | |
| BMI | 1.03 | 0.96 - 1.10 | 0.39 | N/A | 0.97 | 0.84 - 1.12 | 0.70 | 1.05 | 0.97 - 1.13 | 0.22 |
| WHR | 1.02 | 0.96 - 1.08 | 0.58 | N/A | 0.97 | 0.85 - 1.10 | 0.62 | 1.03 | 0.97 - 1.11 | 0.34 |
| WC | 1.01 | 0.94 - 1.09 | 0.79 | N/A | 0.97 | 0.83 - 1.14 | 0.73 | 1.02 | 0.94 - 1.11 | 0.66 |

Odds ratios are given for the effect of each unit increase in standardised genetic liability on stage 2 hypertension. ^a^ Adjusted for age and sex. ^b^ Adjusted for age, sex, smoking status, alcohol status, meat and fish intake, fruit and vegetable intake, and Low-density lipoprotein cholesterol. BMI, Body Mass Index; WHR, Waist Hip Ratio; WC, Waist Circumference; CI, confidence interval. Genetic liability was estimated using African ancestry-derived SNPs on Imputed dataset.

References

1. Winkler TW, Justice AE, Graff M, et al. The Influence of Age and Sex on Genetic Associations with Adult Body Size and Shape: A Large-Scale Genome-Wide Interaction Study. *PLoS Genet*. 2015;11(10):e1005378. doi:10.1371/JOURNAL.PGEN.1005378

2. Shungin D, Winkler T, Croteau-Chonka DC, et al. New genetic loci link adipose and insulin biology to body fat distribution. *Nature 2015 518:7538*. 2015;518(7538):187-196. doi:10.1038/nature14132

3. Ng MCY, Graff M, Lu Y, et al. Discovery and fine-mapping of adiposity loci using high density imputation of genome-wide association studies in individuals of African ancestry: African Ancestry Anthropometry Genetics Consortium. *PLoS Genet*. 2017;13(4):81. doi:10.1371/JOURNAL.PGEN.1006719

4. Liu CT, Monda KL, Taylor KC, et al. Genome-Wide Association of Body Fat Distribution in African Ancestry Populations Suggests New Loci. *PLoS Genet*. 2013;9(8):e1003681. doi:10.1371/JOURNAL.PGEN.1003681
